# Sediment yield estimation and evaluating the best management practices in Nashe watershed, Blue Nile Basin, Ethiopia

**DOI:** 10.1007/s10661-023-11337-z

**Published:** 2023-05-24

**Authors:** Megersa Kebede Leta, Muhammad Waseem, Khawar Rehman, Jens Tränckner

**Affiliations:** 1 Faculty of Agriculture and Environmental Sciences, University of Rostock, 18051 Rostock, Germany; 2 Faculty of Civil and Environmental Engineering, Jimma Institute of Technology, Jimma University, 378 Jimma, Ethiopia; 3 Department of Civil Engineering, Ghulam Ishaq Khan Institute of Engineering Sciences and Technology, Topi, 23640 Pakistan

**Keywords:** Best management practices, MUSLE, Scenarios, Sediment yield, Soil erosion, SWAT

## Abstract

Sediment yield estimation along with identification of soil erosion mechanisms is essential for developing sophisticated management approaches, assessing, and balancing different management scenarios and prioritizing better soil and water conservation planning and management. At the watershed scale, land management practices are commonly utilized to minimize sediment loads. The goal of this research was to estimate sediment yield and prioritize the spatial dispersion of sediment-producing hotspot areas in the Nashe catchment using the Soil and Water Assessment Tool (SWAT). Moreover, to reduce catchment sediment output, this study also aims to assess the effectiveness of certain management practices. For calibration and validation of the model, monthly stream flow and sediment data were utilized. The model performance indicators show good agreement between measured and simulated stream flow and sediment yields. The study examined four best management practice (BMP) scenarios for the catchment’s designated sub-watersheds: S0 (baseline scenario), S1 (filter strip), S2 (stone/soil bunds), S3 (contouring), and S4 (terracing). According to the SWAT model result, the watershed’s mean yearly sediment output was 25.96 t/ha. yr. under baseline circumstances. The model also revealed areas producing the maximum sediment quantities indicating the model’s effectiveness for implementing and evaluating the sensitivity of sediment yield to various management strategies. At the watershed scale, treating the watershed with various management scenarios S1, S2, S3, and S4 decreased average annual sediment yield by 34.88%, 57.98%, 39.55%, and 54.77%, respectively. The implementations of the soil/stone bund and terracing scenarios resulted in the maximum sediment yield reduction. The findings of this study will help policymakers to make better and well-informed decisions regarding suitable land use activities and best management strategies.

## Introduction

The continuous rise in global temperature and precipitation due to land use land cover (LULC) changes has induced an unprecedented increase in soil loss and sediment output (Gashaw et al., 2019). It has been reported that LULC changes are the major contributing factors to reservoir sedimentation. Similarly, the LULC changes increase sediment yield and disturb the water balance and its variability (Wang et al., 2012). Furthermore, inappropriate management and soil conservation strategies lead to excessive erosion of topsoil during heavy precipitations due to which direct runoff increases and infiltration reduces. This causes a further increase in sediment transport and accelerates sedimentation in the reservoir (Ali et al., 2014). Sediment yield and soil erosion dynamics in a given watershed exhibit spatial and temporal variability. The extent of dispersion depends on several factors including land use, vegetation cover, geology, climate, local rainfall patterns, surface runoff, soil class, topographic character, gradient, and drainage parameters. Escalated catchment soil erosion has become serious concern around the globe, especially in developing countries. The contributing factors include agricultural lands, urbanization, deforestation, unsuitable land cultivation as well as cultivation of steep lands and uncontrolled overgrazing without proper management (Abebe & Sewnet, 2014; Kidane & Alemu, 2015; Leta et al., 2021b; Ninija Merina et al., 2016).

In Ethiopian highlands, significant soil erosion has diminished soil fertility and led to siltation in lakes and storage reservoirs. The erosion and siltation processes have impacted the entire Blue Nile River Basin (Megersa et al., 2017; Mhazo et al., 2016). In addition to irreparable environmental impacts in the watershed, the erosion and deposition affect downstream water quality (Ali et al., 2021). It is reported that sedimentation and siltation affects all reservoirs, and while the problem cannot be completely eradicated, it may be managed by various methods on the upstream reservoir catchment (Leta & Chakravarti, 2017). The sediment ladened water is also a greater threat to aquatic life (Annandale et al., 2016; Vaezi et al., 2017). During periods of heavy precipitation, excessive sediment output escalates the risk of flooding, degrades water quality, and reduces the reservoir capacity and sustainability of dams.

Globally, an estimated decrease of 1 to 2% in the existing storage capacity is reported (Zarris & Lykoudi, 2017). Estimation and quantification of sediment yield are very crucial for reservoir sedimentation and river morphology since it helps in planning the reservoir construction capacity to meet the storage requirements throughout the design life. However, due to the unavailability of required data on watershed characteristics, it is a serious challenge in underdeveloped countries. In the case of Ethiopian catchments, the available data on sediment load and rates of accumulation is limited and unreliable. Therefore, the development and application of hydrological models to estimate and evaluate the spatial and temporal dispersion of erosion, sediment load, and reservoir settlement is a suitable alternative.

The commonly employed practices for approximating sediment output from field data are bathymetric surveys, empirical analysis, and plotting sediment rating curves (Annandale et al., 2016). Different hydrological models with varying digress of complexity have been devised as effective and reliable tools to estimate erosion and sediment load. The most used hydrological models are Areal Non-point Source Watershed Environment Response Simulation (ANSWERS), European Soil Erosion Model (EuroSEM), Water Erosion Prediction Project (WEEP), Chemicals, Runoff, and Erosion from Agricultural Management Systems (CREAMS), Agriculture Nonpoint Pollution Source (AGNPS), and Soil and Water Assessment Tool (SWAT). The SWAT is proposed to estimate the effects of various management strategies on water resources, sediment load, and catchment pollution. The SWAT model has previously been widely employed on catchment scale around the world to model and simulate soil erosion under various management scenarios that were affected by the uncertainties of LULC changes into the reservoir (Abdelwahab et al., 2018; Chia & Mbajiorgu, 2018; Leta et al., 2021a).

Similarly, the SWAT model can simulate stream flow, soil loss, sediment output, water quality, and evaluation of best management practices (BMPs) with significant precision and in more detail than many other watershed models in large watersheds on a daily time step (Aouissi et al., 2014; Jha & Gassman, 2014). Therefore, the main advantage of the model is that it performs well and is successful in analyzing the effects of different land management strategies on long-term water and sediment yields within a watershed using easily available data in several validation experiments (Jha & Gassman, 2014). The fundamental issue with the other models is their high number of input parameters and a lack of data to validate model predictions (Annandale et al., 2016; Leta et al., 2021b). The SWAT model efficiently employs the built-in Modified Universal Soil Loss Equation (MUSLE) to predict the watershed soil loss and sediment yield, particularly in catchments where stream flow and sediment data is limited and unreliable (Arnold et al., 2012; Bonumá et al., 2012; Zalaki-badil et al., 2017).

The SWAT model was employed in Ethiopian highlands to assess the effects of certain BMPs on reducing sediment yield in Lake Ziway Basin (Aga et al., 2018), Lake Tana sub-basin (Lemma et al., 2019), Upper Blue Nile Basin (Betrie et al., 2011), and Gumera watershed (Asres & Awulachew, 2010). A review of sediment yield in Ethiopia revealed that most of the previous studies on soil erosion were undertaken in the Northern Ethiopian highlands, with little attention given to the northwestern and southern parts of Ethiopia (Haregeweyn et al., 2015). The Nashe watershed, one of the Blue Nile River’s tributaries, has been endangered by significant soil erosion and its consequences (Gizaw & Kebede, 2019). Leta and Chakravarti (2017) studied sediment yield assessment and mitigation measures in Finchaa watershed, Ethiopia. Ayana et al. (2012) studied sediment yield at the Finchaa watershed using SWAT model and predicted estimated sediment yield. In the entire Nashe watershed, there is still a research gap regarding the estimation of stream flow and sediment yield as well as the prioritization of conservation scenarios. As far as the authors’ knowledge, this is the first study to assess the quantity of watershed sediment yield and to apply several best management strategies utilized to reduce the sediment in the Nashe watershed, Ethiopia.

As a result, this study conducted significant preliminary work for the watershed stream flow and sediment yields. Furthermore, the study attempts to bridge this gap by assessing stream flow and sediment yield using the semi-distributed SWAT and prioritizing conservation scenarios in order to gain a better spatial understanding of the BMPs in the Nashe River watershed. First, the stream flow and sediment data were used to calibrate and subsequently validate the model. Then the sediment reduction efficiency of four independent BMPs was assessed against the baseline condition. The Ethiopian government strongly emphasizes the application of catchment management practices to rehabilitate the degraded portion of the basin (Schmid & Zemadim, 2013). During the current study, the effects of LULC changes on spatial and temporal dispersion of the reservoir siltation and sediment output were evaluated for the Nashe watershed along with the identification of soil erosion and sediment hotspot sub-basins. The study’s findings will ultimately highlight crucial policy measures for the watershed managers and are crucial for demonstrating the optimum sediment management scenarios for effective sediment reductions in the Nashe watershed.

## Materials and methods

### Study area

**Fig. 1:**
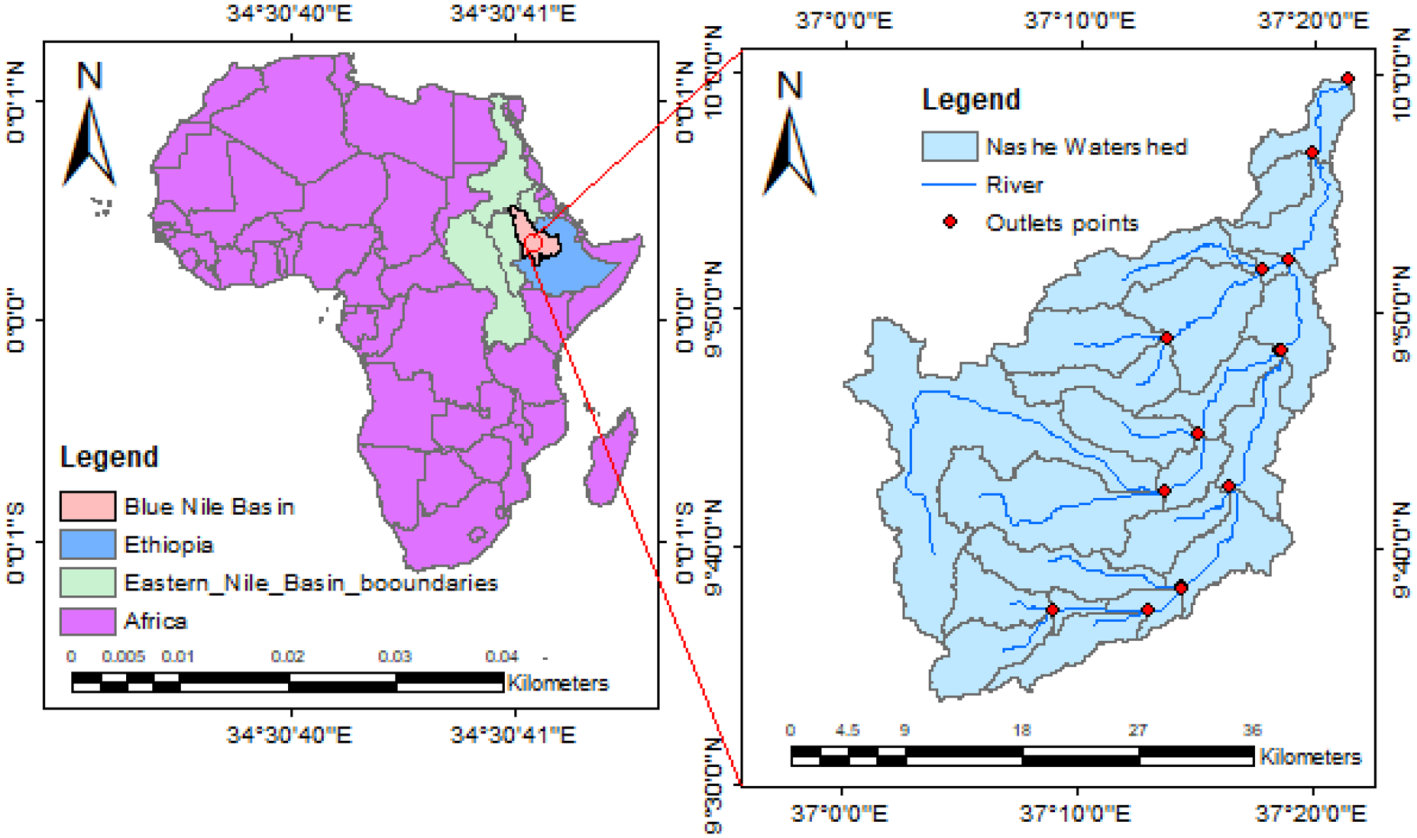
Study area location map

The Blue Nile River Basin emerges at an elevation of 1780 m a.s.l, from Lake Tana in Ethiopia, passing through steep rocky terrain. The basin’s climate is primarily dominated by its geographic position and fluctuates from humid to semi-arid to dry. The Nashe watershed is located 300 km northwest of Ethiopia’s capital, Addis Ababa. The Nashe River emerges from the large extent of a wide river valley with rolling terrain in the Ethiopian highlands, where the Nashe river is isolated through low ridges. Nashe River experiences about 600 m drop from the Nashe cliff into the Amarti River before joining the Finchaa River, which is the Blue Nile River tributary. Nashe River Basin has a subtropical climate with obvious dry and wet seasons. The catchment elevation ranges from 1600 m in the lower flat terrain under the ridge to over 2500 m in the highland hills and ridges. The Nashe dam site is located 450 m upstream of the Nashe fall, where the river channel above the dam site has a length of 53.6 km. The Nashe hydropower reservoir is part of Finchaa Amarti Nashe project (Leta et al., 2022). The sub-basin is located between 9°35′ and 9°52′ N latitudes and 37°00′ and 37°20′E longitudes (Fig. 1). The reservoir sedimentation come from terrestrial erosion of the catchment land and water system transportation. The probable erodibility of the watershed is related to overburdened soil and rainfall intensity.

### Data

The major types of data employed in the modeling framework were soil classes, LULC maps, meteorological, hydrological, and sediment data along with digital elevation model (DEM). The digital elevation model at a spatial resolution of 30 m was acquired with the help of the Shuttle Radar Topography Mission (SRTM). DEM was utilized for the determination of topographic parameters including gradient, the direction of flow, and storage. For the study area, the LULC maps were obtained from Landsat 8 OLI_TIRS scene from the website (https://glovis.usgs.gov/). The maps were processed using supervised classification by ERDAS software. As demonstrated by the data analysis, the major land use land cover is comprised of agricultural, forest, and range lands in the catchment (Leta et al., 2021b).

The soil map of the catchment was acquired from the Ministry of Water, Irrigation, and Energy of Ethiopia (MoWIE). A shape file containing a map of soil classes is created for the SWAT model. The physical and chemical characteristics of the soil were incorporated into a user database to integrate the soil map with the model. The soil map includes 10 types of soil: Haplic Alisols covers the maximum area (47.01%) followed by Rhodic Nitosols (31.77%), Eutric Cambisols, Haplic Arenosols, Chromic Luvisols, Eutric Vertisols, Water, Eutric Leptosols, and Dystric Vertisols. Soil type and characteristics also significantly affect sediment transport. Daily meteorological data from 1985 to 2019 was obtained from the National Meteorological Agency (NMA) of Ethiopia. The dataset included a spatial and temporal variation of rainfall, wind speed, temperature fluctuations over the years, relative humidity, and direct solar radiations. Weather data is one of the most essential data required for hydrological simulations of SWAT. Five weather stations were focused across the study watershed.

**Fig. 2:**
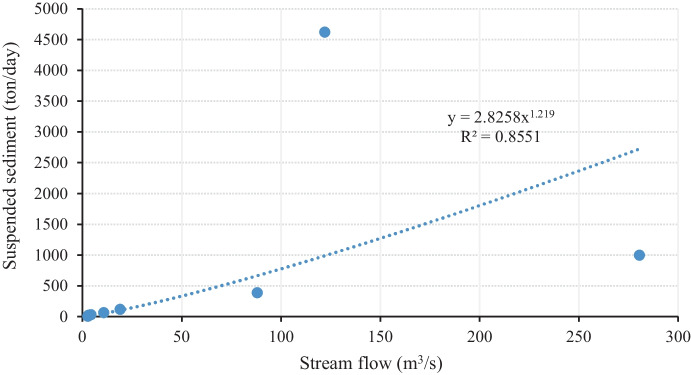
Sediment rating curve of Nashe River

MoWIE also provided the stream flow data of the catchment at the gauging stations from 1985 to 2008. As the sediment data acquired for the Nashe watershed did not have a consistent interval, a sediment rating curve was developed to obtain sediment and flow data in consistent time steps (Fig. 2). The sediment rating curve illustrates a correlation between river discharge and suspended sediment quantity. Sediment rating curve is one of the most applied techniques for evaluating the concentration of suspended sediments delivered by a stream. Firstly, the suspended sediment saturation (mg/l) provided by the MoWIE was converted to sediment load (ton/day) using Eq. 1, then sediment rating curve between the measured sediment load and continuous daily time step stream flow was generated (Eq. 2). The sediment rating curve demonstrates the average correlation of stream flow with sediment quantity in suspension for a given region. The following equation can be used to develop a suspended sediment rating curve based on an average relationship of stream flow with suspended sediment load.

1 $$\mathrm{Qs}={0.0864}^{*}{\mathrm{Sc}}^{*}\mathrm{Q}$$

2 $$\mathrm{Qs}={\mathrm{a}}^{*}{\mathrm{Q}}^{\mathrm{b}}$$

where *Qs* represents sediment quantity in ton/day, *Sc* is the suspended sediment concentration (mg/l), *Q* denotes stream flow in m^3^/s, and *a* and *b* are the regression constants.

## *Model description*

The SWAT is an integrated mathematical model for simulating daily water movement, land cover specification, sediment and nutrient yield and transportation, and quantity of washed-out pesticides from the catchment area under land use land cover dynamics (Farhan & Nawaiseh, 2015). This model was initially developed by the US Department of Agriculture (USDA) to simulate the impacts of various hydrological parameters such as LULC, soil classes, climate, sediment load, pollutants, nutrients, water management strategies, crop yield, and environmental factors (Arnold et al., 2012; Neitsch et al., 2011). The predicted parameters, i.e., stream flow, sediment load, nutrients quantity, and pollutants, are directed through a network of land drains to the catchment outlet (Ayana et al., 2012; Megersa et al., 2017). Modeling the output sediment load includes SWAT project setup, catchment demarcations, analysis of hydrological response units (HRUs), creating and organizing input values, and model simulation.

The SWAT is a combination of continuous catchment simulation models that reduces idealization errors due to lumped, stationary, and linear system assumptions. To reduce the impacts of rainfall variability in space and probable discrepancies in weather input data in determining catchment precipitations from point estimates, the advantage of close vicinity gauges was employed in generating the weather database for SWAT (Habte et al., 2013). Sensitivity analysis determines the sensitivity of output parameters to variation in specified model input parameters. The model parameters having a substantial impact on the calibration of hydrological models for a specific watershed are reduced to the most sensitive types.

In SWAT, multiple sub-catchments are created based on average parameters within a watershed, and then each sub-watershed is classified into one or many HRUs with relatively consistent groups of LULC, soil, and topographic characteristics of the terrain from DEM (Ayana et al., 2012; Megersa et al., 2017). HRU is the most basic unit for the catchment physical process discretized based on LULC variations, soil type, and gradient categories (Leta et al., 2021a). The catchment is defined by the hydrologic process employing the water balance equation (Eq. 3), acting as driving force for flows within the SWAT simulations (Neitsch et al., 2011).

3 $$\mathrm{SWt}=\mathrm{SWo}+\sum\nolimits_{\mathrm{i}=1}^{n}\left(\mathrm{Rday}-\mathrm{QSurf}-\mathrm{Ea}-\mathrm{Wseep}-\mathrm{Qgw}\right)$$

where *SWo* and *SWt* are the initial and final level of water in mm, respectively, *t* represents the duration in days, and *Rday* denotes the precipitation quantity in mm on a given day, *Qsurf* represents the direct runoff quantity in mm on a given day, *Ea* stands for the evapotranspiration quantity in mm on a given day, *Wseep* denotes the quantity of water that seeps from the soil to unsaturated zone on in mm a given day, and *Qgw* represents the quantity of water that returns on a given day in mm.

The sediment load is defined as the gross quantity of sediment eroded by direct runoff, gully erosion, and stream erosion. The erosion severity and sediment output due to precipitation and direct runoff are simulated on daily time step in SWAT using Modified Universal Soil Loss Equation (MUSLE) for each HRU, and the sediment yield is estimated in each sub-catchment (Tuppad et al., 2015) (Eq. 4). MUSLE are used to estimate the quantity of sediments from volume of overland flow, peak surface flow rate, HRU area, erosion capacity of soil, protective techniques, land cover and management strategies, and USLE factors in coarse fragment.

4 $$\mathrm{Sed}=11.8\left(\mathrm{Qsurf}*\mathrm{qpeak}*\mathrm{Areahru}\right)0.56*\mathrm{KUSLE}*\mathrm{PUSLE}*\mathrm{LSUSLE}*\mathrm{CUSLE}*\mathrm{CFRG}$$

where *Sed* represents the sediment quantity in metric tons on a given day, *11.8* is the unit conversion factor, *Qsurf* denotes the overland flow in mm/ha, *qpeak* denotes the peak overland flow rate in m^3^/s, *Areahru* represents the HRU area in ha, *KUSLE* denotes the soil erodibility factor, *CUSLE* is the cover and management factor, *PUSLE* is the support practice factor, *LSUSLE* is the topography factor, while the coarse fragment factor is denoted by *CFRG*.

The catchment hydrology is represented as the land phase in the SWAT model and is routed through main channels using the variable storage technique or Muskingum method (Nguyen et al., 2018). The stream power and peak flow velocity determine the sediment routing in the channel. As a result, SWAT has a built-in algorithm for erosion prediction that takes system’s spatial dispersion into account using data inputs such as soil class, land use, topography, and management practices to estimate watershed erosion and sediment output (Bisantino et al., 2013). Sediment routing in direct runoff and stream flow is simulated depending on precipitation data, topography, soil parameters, and land use land cover. The suspended sediment load in the stream and the transport capacity of the stream determines the probability of settlement in the reach (Neitsch et al., 2011) (Eq. 5). The following equation is used to compute the total sediment quantity in the reach.

5 $${Sed}_{ch}={Sed}_{ch,i}-{Sed}_{dep}+{Sed}_{deg}$$

where *Sed*_ch_ represents the sediment quantity in metric tons, *Sed*_ch,i_ represents the initial sediment quantity in metric tons, *Sed*_dep_ denotes the deposited sediment quantity in metric tons, and *Sed*_deg_ represents the reentering quantity of sediment to the reach segment in metric tons. The following equation (Eq. 6) can be used to determine sediment quantity that is carried out of the reach:

6 $${Sed}_{out}=\mathrm{ Sedch }\times \frac{\mathrm{Vout}}{\mathrm{Vch}}$$

where *Sed*_out_ represents the quantity of sediment at the outlet in metric tons, the suspended sediment load in the stream is denoted by Sed_ch_ (metric tons), *V*_out_ denotes the outflow volume in m^3^ at each interval, and *V*_ch_ represents the water volume of the reach in m^3^.

## Model calibration and validation

The sensitivity analysis was conducted by employing the catchment parametrization results. It aids in the identification of parameters that have a significant impact on stream flow and sedimentation (Shawul et al., 2013). Sensitivity analysis was used to determine the most sensitive stream flow and sediment load factors for model calibration (Table 2). Various rankings were developed for the sensitive parameters impacting stream flow and sediment output. The sensitivity of the model parameters to stream flow and sediment yield in the catchment was evaluated, and the most sensitive parameters with higher *t*-stat values and lower *p* values were taken. Parameters are significant when the absolute *p* value is lower, and *t*-stat value is larger. A higher *p* value indicates that variations in predictor values are unrelated to variation in the response parameters (Abbaspour, 2015). The calibration, validation, and uncertainties of SWAT model prediction were analyzed using SWAT Calibration and Uncertainty Procedures (SWAT-CUP), a package for calibration, validation, and integrated sensitivity analysis through the observed runoff and sediment intensity (Welde, 2016). Calibration and validation are the two fundamental activities in hydrological simulation that must be completed properly before deploying a model. Calibration is a trial-and-error procedure in which simulated data is compared with measured data after each trial through parameter assessment and is used to determine the best appropriate modeling parameter.

Model validation involves the comparison of calibrated model findings with exclusive datasets at various levels of calibrated parameters without additional adjustments for stream flow and sediment production (Neitsch et al., 2011). The flow parameters and sediment data were employed for calibration and subsequent validation of the SWAT model to exhibit the model’s accuracy and effectiveness for estimating stream flow and sediment yield. Furthermore, because during calibration the optimized model parameters are utilized for testing the model without additional modifications, the credibility of available data for calibration is more significant than the validation dataset. The stream flow and sediment yield were subsequently calibrated, as shared transport processes and surface runoff are interdependent which has a direct influence on soil erosion (Arnold et al., 2012). Time series data were utilized to calibrate and validate the model for a period of 23 years (1985–2008). Out of which 1985 and 1986 served as a period for getting the model started. The model was calibrated with available stream flow and sediment discharge data for 12 years (1987–1999). The model was subsequently validated using stream flow and sediment data for 9 years (2000–2008). The SWAT model was calibrated and validated with stream flow data at monthly time-steps, acquired from Nashe stream gauging stations.

Finally, the simulation efficiency of the model was assessed to determine the correspondence between measured and simulated flow parameters and sediment output. Various available indicators for the SWAT model’s performance evaluation and checking the best fit of simulated data with measured monthly data are *r*-factor (zero to infinity), *p*-factor (0 to 100%), Nash–Sutcliffe efficiency (NSE), percent bias (PBIAS), coefficient of determination (*R*^2^), and RSR (the ratio of root mean square error (RMSE) to the standard deviation of measured data), Kling-Gupta efficiency (KGE), and others as depicted in Eqs. 7–13. The calibrated and validated model were checked against the performance statistics ratings (Table 1) for monthly time steps.

7 $$\mathrm{NSE}= 1- \left[\frac{{{\sum }_{\mathrm{i}=1}^{\mathrm{n}}(\mathrm{Qoi}-\mathrm{Qsi})}^{2}}{{{\sum }_{\mathrm{i}=1}^{\mathrm{n}}(\mathrm{Qoi}-\overline{\mathrm{Q}}\mathrm{o })}^{2}}\right]$$

8 $$\mathrm{PBIAS}= 100* \left[\frac{{\sum }_{\mathrm{i}=1}^{\mathrm{n}}(\mathrm{Qoi}-\mathrm{Qsi})}{\sum_{\mathrm{i}=1}^{\mathrm{n}}\mathrm{Qoi}}\right]$$

9 $${\mathrm{R}}^{2}=\frac{{\left[{\sum }_{\mathrm{i}=1}^{\mathrm{n}}(\mathrm{Qsi}-\overline{\mathrm{Q}}\mathrm{s })(\mathrm{Qoi}-\overline{\mathrm{Q}}\mathrm{o })\right]}^{2}}{{{\sum }_{\mathrm{i}=1}^{\mathrm{n}}(\mathrm{Qsi}-\overline{\mathrm{Q}}\mathrm{s })}^{2}{{\sum }_{\mathrm{i}=1}^{\mathrm{n}}(\mathrm{Qoi}-\overline{\mathrm{Q}}\mathrm{o })}^{2}}$$

10 $$\mathrm{RSR}= \frac{\mathrm{RMSE}}{\mathrm{STDEVob}}= \frac{\sqrt{\sum_{\mathrm{i}=1}^{\mathrm{n}}{(\mathrm{Qoi}-\mathrm{Qsi})}^{2}}}{\sqrt{\sum_{\mathrm{i}=1}^{\mathrm{n}}{(\mathrm{Qoi}-\overline{\mathrm{Q}}\mathrm{si })}^{2}}}$$

11 $$\mathrm{bR}^2=\mathrm{Maximize}:\Phi=\left\{\begin{array}{c}\left|\mathrm b\right|\mathrm R^2if\left|\mathrm b\right|\leq1,\\\left|\mathrm b\right|^{-1}\mathrm R^2if\left|\mathrm b\right|>1\end{array}\right.$$

12 $$\mathrm{SSQR}=\frac{1}{\mathrm{n}}{\sum }_{\mathrm{i}=1}^{\mathrm{n}}{\left(\mathrm{Qoj}-\mathrm{Qsj}\right)}^{2}$$

13 $$\mathrm{KGE}=1- \sqrt{{\left(\mathrm{r}-1\right)}^{2}+ {\left(\mathrm{\alpha }-1\right)}^{2}+{\left(\upbeta -1\right)}^{2} }$$

where *Q*_si_ and *Q*_oi_ denote the simulated and actual stream flow values in m^3^/s, $\overline{Q }o$ and $\overline{Q }s$ represent the average values of the actual and simulated stream flow respectively in m^3^/s, *b* represents the regression line slope between the simulated and measured variables, *j* represents the rank, *r* denotes the linear regression coefficient between the measured and simulated variables, $\upbeta =\frac{\mathrm{\mu s}}{\mathrm{\mu m}}$ and $\mathrm{\alpha }=\frac{\mathrm{\sigma s}}{\mathrm{\sigma m}}$, *µs* and *µm* represent the average of simulated and measured parameters, respectively, *σs* and *σm* denote the standard deviations of the simulated and observed data, respectively.

**Table 1:** Flow and sediment yield estimation utilizing model performance and classical objective functions

	Statistical efficiency criterion		Model performance ratings	
Objective function	Characteristics	Function category	Value range	Performance rating
NSE	Most common; emphasize on high flows; neglect the low flows	Distance-based	NSE ≤ 0.5 0.5 < NSE ≤ 0.65 0.65 < NSE ≤ 0.75 0.75 < NSE ≤ 1	Unsatisfactory Satisfactory Good Very good
RSR	Monotony; cannot be used alone	Distance-based	RSR > 0.7 0.6 < RSR ≤ 0.7 0.5 < NSE ≤ 0.6 0.5 < NSE ≤ 0	Unsatisfactory Satisfactory Good Very good
PBIAS	Monotony; cannot be used alone	Weak form-based	PBIAS ≥ ± 25 ± 15 ≤ PBIAS < ± 25 ± 10 ≤ PBIAS < ± 15 PBIAS < ± 10	Unsatisfactory Satisfactory Good Very good
*R*^2^	Emphasize on high flows	Weak form-based	*R*^2^ < 0.50 0.50 < *R*^2^ < 0.70 0.70 < *R*^2^ < 0.80 > 0.80	Unsatisfactory Satisfactory Good Very good

### Watershed best management practices

In this work, the BMPs adopted in various land management scenarios were analyzed based on which managers can devise conservation techniques having the highest efficiency for the Nashe watershed. The execution of best management practices (BMPs) in catchments (critical sub-catchments) has been identified as an efficient technique to significantly reduce soil loss and sediment concentration. Depending on the spatial dispersion of sediment yield, the source level recognized by the SWAT model was employed for predicting the efficiency of BMPs. In many parts of the globe, the SWAT hydrological model is extensively used to assess the efficiency of BMPs in terms of reducing agricultural non-point source pollution and sediment yield (Briak et al., 2019; Kumar et al., 2019; López-Ballesteros et al., 2019; Merriman et al., 2019). Watershed management plans have a significant influence on reducing catchment soil erosion by implementing appropriate management strategies (Abdelwahab et al., 2014).

The choice of BMP scenarios and related parametric values specific to a given region should represent its land use practices, and should be based on traditional conservation techniques for land and water in Ethiopian highlands (Abdelwahab et al., 2014). The management scenarios were constructed based on erosion intensity, overland flow (hotspot locations), and the most sensitive simulation parameters. Gashaw et al. (2021)assessed the erosion reduction efficiency of best management practices (BMPs) in Gumera catchment of the Upper Blue Nile watershed using the SWAT model. During the current study, the impacts of BMPs on sediment output were simulated using the SWAT model, and reservoir conservation techniques were prioritized based on sediment reduction efficiency and performance.

The SWAT model was used to predict the influence of best management practices on sediment control, identify maximum sediment yield hotspots, and implement management practices to reduce sediment yield. Four management scenarios were chosen for this study, all with independent impacts on flow and sediment characteristics. The four management methods were implemented in sub-basins with high sediment production. These BMPs were modeled by adjusting the relevant SWAT parameters to study the impacts of the alternative practices on the estimated sediment yield. The following scenarios were simulated in SWAT: baseline (S0), filter strip (S1), stone/ soil bunds (S2), contouring (S3), and terracing (S4).

The baseline scenario (S0) describes the catchment in the existing condition and serves as a benchmark for the simulation of alternative management scenarios. The filter strip scenario (S1) is explored because vegetation along farmland contours, like grasses reduces overland flow and sheet and rill scour, increases infiltration and base flow, and enhances sediment entrapment (Betrie et al., 2011). At the HRU level, soil type and land use arrangement may add a significant quantity of sediment the o average watershed output. For expressing the filter strip effect, the filter strip width is a suitable model parameter (FILTERW). For evaluating the impacts of the filter strip scenario on sediment abstraction, the filter width of unit value (FILTERW = 1 m) was considered for analysis. The strategy of applying stone/soil bunds (S2) to reduce erosion and sediment yield was recognized as a good practice in the Ethiopian highlands. Application of stone/soil bunds reduce overland flow and sediment loss by shortening slopes and increasing watershed abstractions (Addis et al., 2016). This strategy aims to decrease runoff, sheet scour, and slope length. Modifying parameters such as the length of slope (SLSUBBSN), steepness of slope (HRU_SLP), soil conservation services (SCS) curve number (CN2), and the erosion control practice factor (USLE_P) for key sub-basins simulate the impacts of stone/soil bunds creation on steep grades (Ashrafi et al., 2017; Kassawmar et al., 2018).

According to Ebabu et al. (2018), one of the most efficient management techniques to minimize direct runoff and soil loss in the Upper Blue Nile Basin is the application of soil/stone bunds reinforced with grass. During periods of excessive precipitation, contour lines (S3) build a water break, reducing the creation of rills and gullies. This conservation technique in SWAT model was reflected by adjusting CN2 and the corresponding USLE_P, which is a ratio that compares soil loss from one support system to soil loss from up and down cultivation. Terracing (S4) acts as a runoff barrier, enhancing infiltration while reducing runoff. It consequently attenuates the erosion power of overland flow and induces siltation. This strategy raises the available quantity of water to recharge aquifers at low depths. By modifying the values of appropriate factors like the CN2, USLE_P, and SLSUBBSN, the terracing scenario was incorporated. Depending on slope classes the SLSUBBSN value is modified. Suitable curve number and USLE_P were determined based on soil class, gradient, and land use land cover (Arnold et al., 2012).

## Results and discussions 

### The stream flow sensitivity and sediment parameters

The most sensitive variables with required accuracy for calibration and the sensitivity level of flow and sediment variables are given in Table 2 and Table 3, respectively. The *t*-stat and *p* values statistical criteria are employed to evaluate the relative importance and sensitivity of individual parameters. Accordingly, the most top 5 sensitive parameters for stream flow were SCS runoff curve number (r__CN2.mgt), Ground water Delay from soil to channels(days) (v__GW_DELAY.gw), Saturated hydraulic conductivity (mm/hour) (r__SOL_K (..).sol), Base flow alpha factor for bank storage (v__ALPHA_BF.gw), and Manning’s roughness coefficient (main channel) (v__CH_N2.rte).

**Table 2:** The sequence of the parameters used in stream flow calibrations, as well as the fitted sensitivity values of stream flow

Parameter name	Descriptions	Sensitivity			Parameter value		
		t-stat	*P* value	Rank	Min	Max	Fitted
r__RCHRG_DP.gw	Deep aquifer percolation fraction	− 0.23	0.41	9	0	1	0.819
r__SLSUBBSN.hru	Average length of slope (m)	− 0.39	0.32	8	0	150	73.98
r__SOL_AWC (..).sol	Soil existing water capacity (mm H_2_0/ mm soil)	− 0.47	0.64	7	− 25	25	− 12.1
v__GWQMN.gw	Critical water depth in shallow aquifer allowing back flow(mm)	2.18	0.01	6	0	5000	1795
v__CH_N2.rte	Manning’s roughness coefficient (main channel)	− 2.81	0.27	5	0	1	0.393
v__ALPHA_BF.gw	Base flow alpha factor for bank storage	3.14	0.03	4	0	1	0.367
r__SOL_K (..).sol	Saturated hydraulic conductivity (mm/hour)	− 8.30	0.00	3	− 25	25	19.05
v__GW_DELAY.gw	Ground water Delay from soil to channels (days)	7.25	0.00	2	0	500	19.5
r__CN2.mgt	SCS runoff curve number	13.33	0.00	1	− 25	25	− 13.1

**Table 3:** The sequence of the parameters used in sediment calibrations, as well as the fitted sensitivity values of sediment yield

Parameter name	Descriptions	Sensitivity			Parameter value		
		t-stat	*P* value	Rank	Min	Max	Fitted
V__CH_COV2.rte	Channel cover factor	0.03	0.63	7	0.001	1.0	0.87
V__PSP.bsn	Sediment routing factor in main channel	− 0.51	0.41	6	0.0	1.0	0.89
V__CH_COV1.rte	Channel erodibility factor	− 0.63	0.35	5	0.01	0.6	0.50
V__SPCON.bsn	Linear re‐entrainment parameter in sediment routing	0.84	0.03	4	0.0001	0.01	0.001
V__LAT_SED.hru	Sediment concentration in lateral and groundwater flow (mg L^−1^)	− 1.21	0.02	3	0.0	1000	530.00
V__SPEXP.bsn	Exponential factor for sediment routing	2.08	0.00	2	1.0	2.0	1.19
V__USLE_P.mgt	USLE support practice factor	− 25.99	0.00	1	0.0	1.0	0.03

The most sensitive parameters for sediment parameters were USLE support practice factor (V__USLE_P.mgt), Exponential factor for sediment routing (V__SPEXP.bsn), Sediment concentration in lateral and groundwater flow (mg L − 1) (V__LAT_SED.hru), Linear re‐entrainment parameter in sediment routing (V__SPCON.bsn), and Channel erodibility factor (V__CH_COV1.rte). The recommended ranges indicated in Table 2 and Table 3 were utilized to calibrate these parameters, which were then used to estimate the rate of soil erosion from the watershed and the channel.

For the convenience of the calibration process, the highly effective flow parameters and sediment output variables are identified. The initial effective sediment parameters were calibrated through a universal sensitivity analysis approach through which the variation rate in model output parameters upon adjustment of model input parameter was evaluated. The size and spatial variability of watershed sediment output were also assessed. Therefore, the most sensitive variables were calibrated over a specified range throughout the entire calibration period. In this study, the CN2 and USLE P parameters were singled out as most sensitive to stream flow and sediment output, respectively.

### Performance evaluation, calibration, and validation

**Fig. 3:**
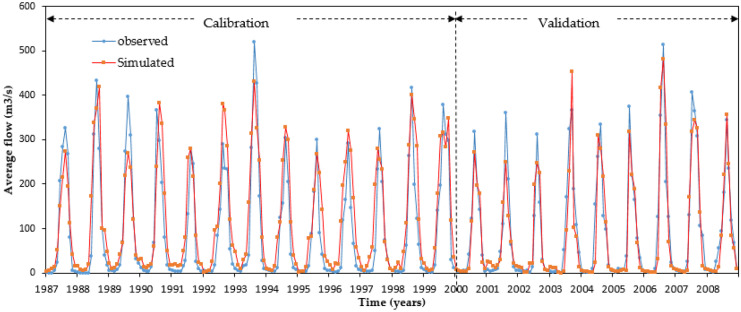
Comparison of measured and simulated monthly runoff hydrograph for calibration and validation phases

**Fig. 4:**
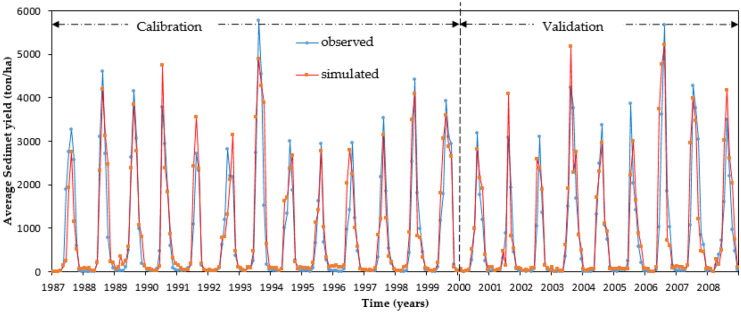
Monthly calibration and validation outcomes of measured and simulated sediment output

For the stream flow data, the model was calibrated first as recommended by Arnold et al. (2012) followed by calibration for sediment yield-related parameters. The goal of the calibration method was to make the uncertainty band as low as possible while bracketing most of the data within the 95PPU. Various calibration settings were employed for each HRU to generate weighted mean results for each sub-catchment. Figure 3 depicts the agreement of calibration period of measured stream flow data with simulated measurements through perfect match line. The simulated and measured sediment yield for their corresponding calibration and validation phases are exhibited in Fig. 4. During the calibration and validation phase, acceptable results were obtained for stream flow and sediment yield using SWAT model (Tables 4 and 5).

**Table 4:** Measured and simulated average stream flow calibration and validation data

Monthly simulation	*R*^2^	NSE	PBIAS	RSR	*P*-factor	*r*-factor
Calibration	0.81	0.79	− 5.9	0.7	0.75	0.64
Validation	0.84	0.81	3.7	0.5	0.81	0.68

**Table 5:** Monthly observed and simulated sediment yield calibration and validation statistics

Monthly simulation	*R*^2^	NSE	PBIAS	RSR	*P*-factor	*r*-factor
Calibration	0.76	0.75	− 19	0.5	0.65	0.54
Validation	0.79	0.81	6.8	0.4	0.69	0.66

Using the model performance assessment criteria, the simulated and observed monthly stream flow had good correspondence. The model’s eligibility for scenario analysis was confirmed by its increased performance during the validation phase. The PBIAS, *R*^2^, and NSE were − 5.9%, 0.81, and 0.79 for the calibration period and 3.7%, 0.84, and 0.81 for the validation phases, respectively. From the model’s performance grading criteria, *R*^2^ and NSE for calibration were 0.76 and 0.75, while for validation phase 0.79 and 0.81 for measured and simulated monthly average sediment output respectively (Table 5).

During the model calibration phase, comparatively low statistical measures (NSE and *R*^2^) may be due to authenticity and unavailability of data, overland flows, and simulation idealizations in employing measured data obtained from the rating curve equation, discrepancies in peak flow approximation, and model simulations. The overall performance of stream flow and sediment output simulations during the calibration and validation phase was good with *R*^2^, NSE, and PBIAS values, respectively (Moriasi et al., 2007).

The positive and negative PBIAS values revealed that the stream flow and sediment output is slightly underestimated and overestimated, respectively. During the calibration phase, the measured rate of stream flow was somewhat overestimated by the model, while it was slightly underestimated during the validation phase in the Nashe watershed (Table 3 and Fig. 3). Overall, the PBIAS value suggested that the SWAT model is quite good at simulating sediment yield. The graphical analysis of the simulated and measured sediment yield indicated both overestimation and underestimation of sediment yield during calibration and validation phases, respectively (Table 4 and Fig. 4). Generally, most of the model assessment metrics (coefficients of determination and Nash–Sutcliffe efficiencies) satisfied the assessment criteria for model performance. It shows that good results were obtained for stream flow with SWAT simulations; hence, it can be used to simulate the stream flow satisfactorily.

The observed sediment yield was greater than the simulated mean monthly sediment yield for the Nashe watershed. Due to the reservoir barrier, the sediment carried by overland flows settled in the reservoir, causing sedimentation over time. During periods of heavy precipitation in the Ethiopian highlands, sediment yield is greatly unreliable (Dile et al., 2016; Sultan et al., 2018).

### Sediment yield assessment of the study area watershed

**Fig. 5:**
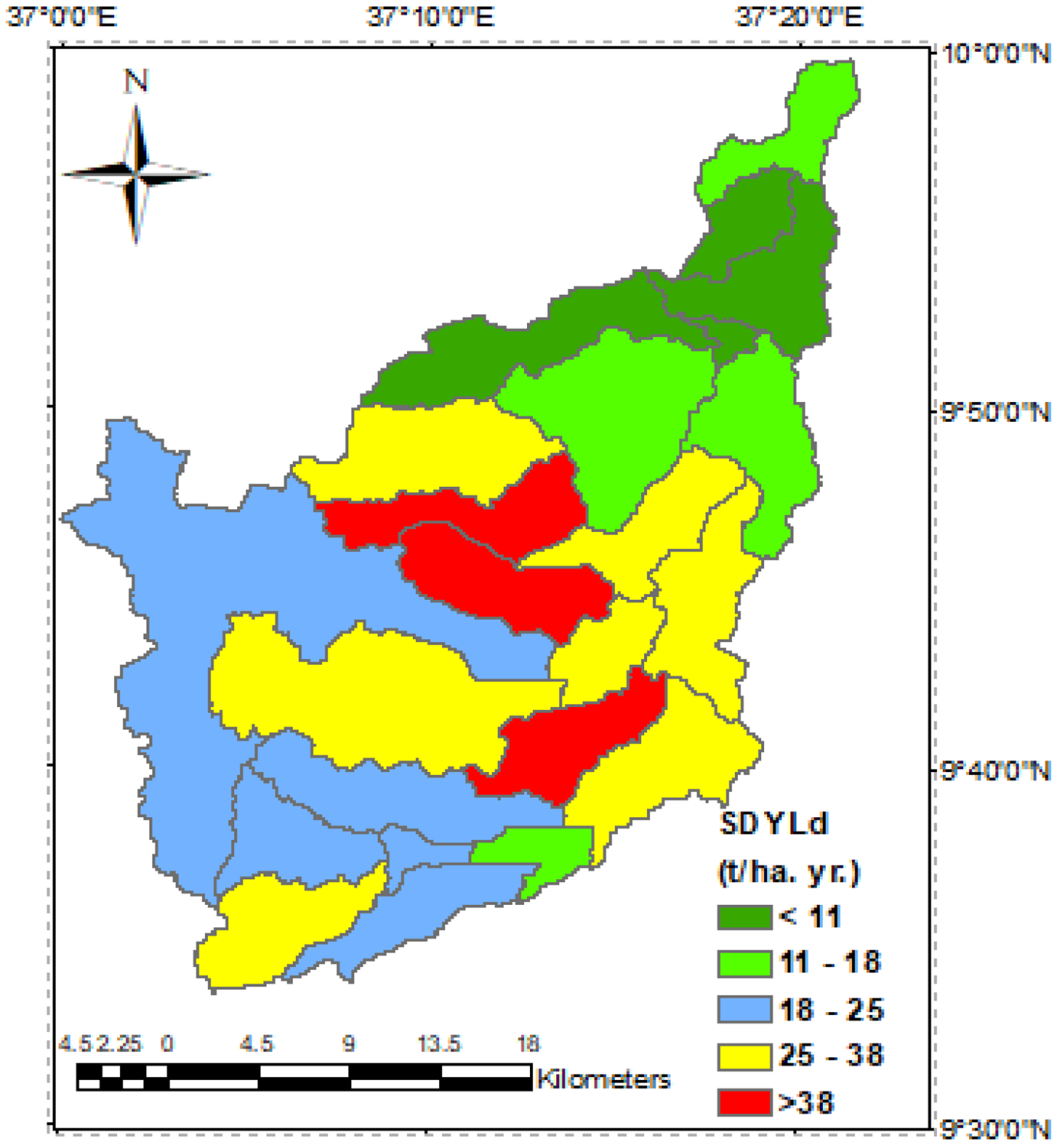
The sediment yields spatial distribution of the watershed

The soil erosion hotspot locations were mapped using estimated average annual sediment yield of the watershed. It is helpful to prioritize locations for planning and choosing land management actions based on differences in the level of erosion risk among sub-basins. The total simulated sediment output of the sub-basins is reported for the whole Nashe watershed, which comprises of 23 sub-basins (Fig. 5). The total quantity of sediment produced in the catchment ranges from minimal to approximately 65.57 t/ha. yr., according to sediment simulations. Sub-basins 2, 3, 4, and 5 had the lowest sediment output. The highest sediment production was found in sub-watersheds 9, 11, and 14, with values ranging from 54.29 to 65.57 t /ha. yr., indicating more erosion exposure. These sub-watersheds account for just 10.77% of the overall area; however, they contribute disproportionately to the highest quantity of sediment loss, accounting for around 30.06% of entire sediment production. Soil erosion has been recognized as the most severe in these sub-basins.

Figure 5 shows the sediment yield dispersion for the catchment as estimated by the model. The sub-watersheds that produce substantial sediment yields can be identified from the figure. The areas having good vegetation cover were associated with the lowest sediment yield. Meanwhile, the highly cultivated fields with hilly and rolling terrain were characterized by high sediment yield and associated with the highest surface runoff. The average sediment output and erosion hotspot regions of the sub-basins are shown to be strongly connected to slope gradient, farmland percentage, and rainfall quantity. Excessive sediment output was found in steep sub-catchments with vast agricultural land and heavy precipitation. The erosion risk is normally modest on slopes less than 8%; however, it escalates as the slope steepens, especially on agricultural land.

**Fig. 6:**
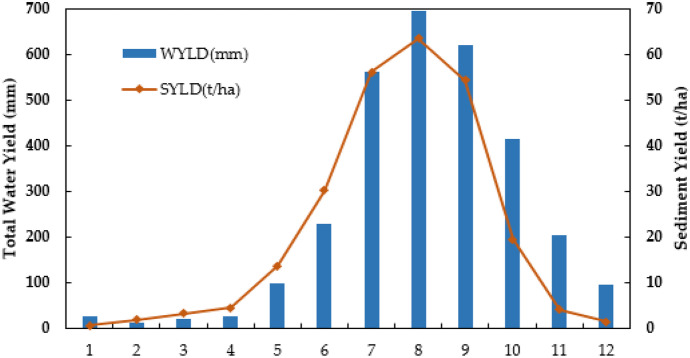
Sediment yield and water yield flow pattern

In addition, the sediment yield was linked primarily to land uses, precipitation, gradient, and soil classes to determine the main source sites as indicated by the model in the HRU study. Because of the spatial variation in key landscape datasets, types of soil, and land use in every HRU, the SWAT model simulations demonstrated a difference in sediment yield from HRU to HRU. Land cover, in addition to gradient and rainfall, is a highly important component (Licciardello et al., 2017) at the level of HRUs in determining whether regions within sub basins have a high risk of erosion. Another research conducted at the Yezet Watershed in northern Ethiopia by Lemlem et al. (2017) found a substantial correlation between slope and sediment output. Based on the model results, a substantial soil erosion risk was estimated around the Northwestern part of the catchment area, the upstream and central part of the watershed. The severity of the sediment yield (Table 6), on the other hand, varies across a region. Sediment yield pattern at the catchment outlet observes stream flow pattern (Fig. 6).

**Table 6:** Severity classification of soil erosion

Sediment yield (t/ha. yr)	Severity classes
< 11	Low
11–18	Moderate
18–25	High
25–38	Very high
> 38	Severe

Furthermore, they are valuable instruments for prioritizing the execution of BMPs in catchment areas (Betrie et al., 2011). According to the models, the highest sediment loads are recorded in July, August, and September. This three-month period accounted for about 68.77% of the entire yearly sediment load. The seasonal fluctuations in the catchment reveal a considerable sediment output influencing the watershed’s surface throughout the summer season (June to September) caused by rainfall events. Meanwhile, during the dry season (October to January), sediment yield is almost negligible. The first stage in mitigating soil erosion is to evaluate the existing condition of the catchment and implement watershed interventions. Prioritizing and implementing catchment management choices require identifying key sub-basins.

Reservoir deposition and sedimentation can be avoided by applying sustainable sediment management (Schleiss et al., 2016). Developing watershed management methods that effectively employ land resources while maintaining environmental quality requires evaluation of the spatial diversity of soil loss (Betrie et al., 2011). Recognizing the spatial dispersion of erosion hotspots is critical for catchment management planning, as it assists in categorizing the necessity of land management intervention. This regional diversity in sediment output throughout the watershed is helpful in prioritizing, selecting, and executing appropriate land management initiatives. As a result, BMPs must be addressed by policymakers at regional and national levels to limit the agricultural impacts on soil depletion.

### The impact of best management practices on sediment yield

**Fig. 7:**
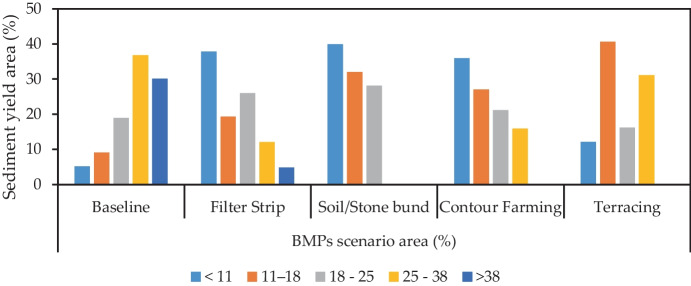
Sediment yield class and percentage of erosion potential areas in each scenario

**Fig. 8:**
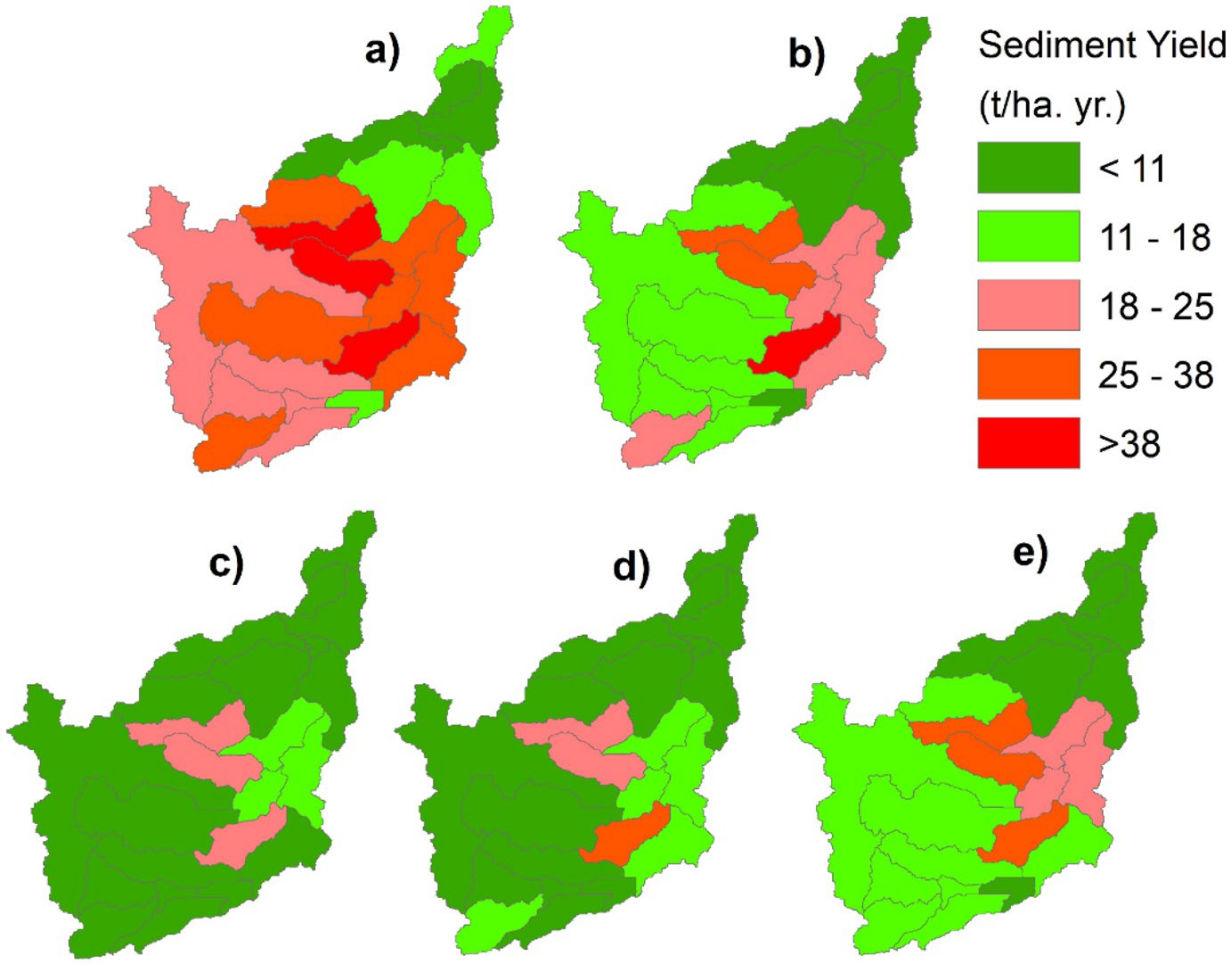
Alternative management scenarios' percent reductions in simulated sediment at the sub-watershed level. Note. **a** base period, **b** filter strip, **c** stone/soil bunds, **d** contouring, **e** terracing

The implementation of various BMPs yielded considerable improvement for sediment yield reduction. The sediment output reduction by implementing conservation techniques was compared with the model outcomes in the current conditions (baseline scenario) as shown in Figs. 7 and 8. Therefore, when compared to the baseline scenario, the imposed watershed management alternatives (filter strips, soil/stone bunds, contouring, and terracing) had a significant impact on the watershed’s sediment output reduction. In the Nashe watershed, 30.06% area is classified as extremely erosion prone, 36.74% and 18.90% as very high and high erosion prone, 9.10% as moderately prone to erosion, and 5.20% as less prone to erosion (Table 7 and Fig. 8). However, by applying best management techniques in the watershed, the percentage of region categorized as high to severe erosion prone was substantially reduced. Because the precipitation, terrain, and percent of agricultural coverage are varied in the Nashe watershed, the influence of each best management practice scenario in sediment reductions is also varied. In general, topographical factors, the proportion of available land, and the kind of land use land cover in chosen sub-watershed all highly influence the efficiency of every conservation measure.

**Table 7:** Sediment yield class and percentage of erosion potential areas in each scenario

SDyld (t/ha. yr)	BMPs scenario area (%)					Severity classes
	Baseline	Filter Strip	Soil/stone bund	Contouring	Terracing	
< 11	5.20	37.88	39.86	35.94	12.10	Low
11–18	9.10	19.32	32.02	27.04	40.61	Moderate
18–25	18.90	25.96	28.11	21.14	16.19	High
25–38	36.74	12.07	0.00	15.88	31.10	Very high
> 38	30.06	4.77	0.00	0.00	0.00	Severe

The average sediment output was reduced by 37.88% in the filter strip scenario (S1) compared to the current condition (S0) at the same outlet point. Manawko (2017) found that average annual sediment yield was reduced by 25.8% filter strips were applied to the proposed middle Awash Dam watershed in Ethiopia. Betrie et al. (2011) also reported that sediment yield reduction ranges from 29 to 68% due to the application of filter strips at Upper Blue Nile. Terracing is a resource management technique that involves decreasing the slope length and gradient of sub catchments to minimize soil loss and sediment output. The sediment yield was reduced by 54.77% in simulations of terracing management options on selected key sub-basins. Therefore, terracing can be used in watershed management to minimize overland flow and soil erosion significantly, to minimize sediment yield (Shao et al., 2013). The study supported previous sediment yield values (Gashaw et al., 2019).

According to Tesfu (2015), sediment yield was reduced by 73.11% after the application of terracing to the crucially affected sub catchments at the Kesem dam catchment, Awash basin, Ethiopia. The simulations of contouring on critical sub-catchments reduced yearly sediment output by 39.55%. The scenario of soil/stone bund reduced the sediment output to 15.254 t/ha. yr, equivalent to 57.98% of the current sediment yield. The simulation results show that the introduction of a soil/stone bund scenario results in the greatest improvements. The sediment yield reduction by soil/stone bund was comparable with the sediment yield reduction reported by Gebrernichael et al. (2005) in field-scale. According to Lemma et al. (2019), the filter strip application to Lake Tana Basin reduced sediment yield by 61%.

Employing the soil/stone bund management strategy results in maximum sediment reduction in key sub-catchments along with catchment-level followed by terracing in the Nashe watershed as shown in Table 7 and Fig. 7. For the same implementation area, the terracing scenario exhibited higher sediment reduction than the contouring scenario. It can be observed that terracing is more effective than contouring. Hence, upon comparison with the baseline scenario, it was observed that among all the BMPs, the filter strip is the least efficient conservation strategy for sediment reduction. According to the study findings, steep agricultural fields are the greatest contributors to erosion and sediment outputs. The steep slope and larger percentage of cultivated land are responsible for areas with high to severe sediment yield classifications.

As presented in Fig. 8, soil erosion risk is lower in areas with a good natural vegetation cover downstream of the catchment. A high risk of soil erosion was estimated around the upstream and central part of the watershed. The high risk of sediment yield on the upstream highlands of the catchment could be related to the uncontrolled community activities on the upstream areas and transformation of natural vegetation into agricultural lands. Slope classes of 15–30 and > 30% encompass roughly 60–80% of the sub watersheds in those sediment severity classes (Table 6). Several researchers have also found that agricultural land expansion results in higher sediment output than other land use land cover changes (Bathrellos et al., 2017; Welde, 2016).

Previous investigations in the country highlands, such as Gashaw et al. (2019) in the Andassa watershed, Setegn et al. (2010) in the Anjeni-gauged watershed, and Tamene et al. (2017) in the Laelaywukro catchment, have all shown similar mean sediment yield. Demissie et al. (2013) in the Gilgel Gibe Basin found a significant rate of soil erosion in sub-catchments where dry land, crops, and pasture covered a major portion of land approximately 60%. At the catchment and sub-catchment level, the idea of assessing the efficiency of specific BMPs can aid in the identification of effective BMPs that can decrease agricultural nutrients and enhance availability of water resource in times of LULC shift, ensuring sustainable water resource management. Therefore, implementing sustainable sediment management will prevent reservoir siltation and sedimentation. The results will be valuable to policymakers, practitioners, and water resource engineers in implementing the most efficient BMPs in the catchment or in other similar catchments.

## Conclusions

In this study, SWAT model was utilized to examine the spatial and temporal dispersion of sediment output in the Nashe watershed. The effects of several best management practices on minimizing watershed sediment yield were also assessed. The most effective variables were identified to calibrate and subsequently validate the model using sensitivity analysis. The actual stream flow and sediment data was used to calibrate and validate the model for simulating sediment yield and management alternatives. The calibration and validation of the model indicated good correspondence between observed and simulated parameters (i.e., stream flow and sediment yield), as evidenced by appropriate NSE, PBIAS, and *R*^2^, values. Model’s statistical performance evaluation criteria for both flow and sediment simulations were within acceptable limits. The Nashe watershed’s annual soil loss estimates were categorized into five erosion severity classes. Prioritization allowed us to identify sub-watersheds in the catchment that are highly susceptible to erosion.

The results revealed that at the baseline condition, 30.06% of the area in the Nashe watershed is identified as severely prone to erosion and 36.74% and 18.90% as very high and high erosion prone, respectively. The simulation findings demonstrate that land conservation strategies could minimize sediment yield by 34–58% at the catchment level. BMP implementation decreased sediment production in areas with very high, high, and moderate severity ratings. BMP adoption, on the other hand, increased regions with low and very low sediment production, indicating that the BMPs accounted for reduced sediment formation in the catchment. Generally, the four management scenarios lead to a significant reduction in sediment yield, and the soil/stone bund (57.98%) had the highest efficiency for sediment output reduction, followed by the terracing scenario (54.77%). The filter strip had the lowest decrease in sediment output. Considering the research findings, it is recommended that the soil/stone bund scenario be implemented in the Nashe watershed for efficient sediment reduction. Areas having steep slopes and substantial cultivation have a higher risk of erosion, classified as severe and extremely high.

In fact, the efficiency of each scenario is heavily influenced by topographical factors and land cover types. The steep slopes and extensive agricultural land induce the high risk of soil erosion, classified as severe and very high. In general, the study found that prioritization is critical not only for identifying erosion hotspots, but also the most effective BMPs. The differences in erosion risk across sub-basins assist planners in identifying and prioritizing specific portions of the catchment that require immediate soil conservation measures. Decision-makers may find the modeling technique useful in determining probable soil erosion causes and subsequent prioritization. The findings of this study will help policymakers make land and water management decisions and can be used as a model for land use implementation in other watersheds. In the Ethiopian highlands and other similar agroecological zones across the world, the simulated effective BMPs can be employed to avoid soil erosion and related negative externalities.

## Data Availability

The datasets generated and/or analyzed during the current study are not publicly available due to privacy reasons but are available from the corresponding author on reasonable request.
